# Case Report: Severe multi-organ damage induced by cadonilimab: mechanism investigation and management strategies

**DOI:** 10.3389/fimmu.2026.1961670

**Published:** 2026-10-01

**Authors:** Bin Shan, Ruixia Yang, Yi Peng, Mengjiao Li

**Affiliations:** 1 Department of Pharmacy, the Fourth Hospital of Hebei Medical University, Shijiazhuang, Hebei, China; 2 Department of Pharmacy, the Second Hospital of Hebei Medical University, Shijiazhuang, Hebei, China

**Keywords:** adverse reactions, bispecific antibody, cadonilimab, immune checkpoint inhibitor, mechanism investigation, multi-organ damage

## Abstract

Most immune checkpoint inhibitors currently used in clinical practice, such as programmed cell death protein 1/programmed death ligand 1 (PD 1/PD L1) inhibitors and cytotoxic T lymphocyte antigen 4 (CTLA 4) inhibitors, are monospecific antibodies acting on a single target. In contrast, bispecific antibodies (BsAbs) contain an additional specific antigen binding site, enabling dual signaling pathway blockade and more potent inhibition of tumor growth. In June 2022, the first PD 1/CTLA 4 BsAb cadonilimab (AK104) was approved by the National Medical Products Administration of China for the treatment of cervical cancer and gastric or gastroesophageal junction adenocarcinoma. Owing to its recent market approval, only a limited number of studies have reported immune related adverse events (irAEs) associated with this drug, involving the skin, gastrointestinal tract, liver, and other organs. Thus far, no case of cadonilimab related multi organ injury has been described. In this article, we report the first case of severe immune related multi organ and endocrine system injury induced by cadonilimab in the treatment of cervical cancer. In addition, we conducted a retrospective literature analysis comparing the adverse drug reaction (ADR) profiles of immune checkpoint inhibitor monospecific antibodies and BsAbs. The aim of this analysis was to provide a reference for the rational use of cadonilimab. Early recognition of irAEs, identification of risk factors, regular monitoring, use of corticosteroids, and supportive care are critical for improving clinical outcomes. It should be emphasized that the oncologic benefits of BsAb therapy generally outweigh the risks of ADRs, and treatment should not be discontinued or delayed except in rare circumstances (e.g., multi organ failure). In case of ADR occurrence, familiarity of the healthcare professionals with the relevant irAEs is essential to facilitate effective diagnosis and appropriate management of this increasingly common pattern of multi organ injury.

## 1 Introduction

Immunotherapy represents the most promising systemic modality for the treatment of cancer and is critically important for improving therapeutic outcomes. The occurrence and progression of malignant tumors involve complex pathohistological and molecular biological mechanisms, and monoclonal antibodies targeting a single antigen are often insufficient to achieve adequate therapeutic effects. This has led to the development of bispecific antibodies (BsAbs), which are artificial antibodies generated through technologies such as cell fusion, recombinant DNA, and protein engineering. BsAbs can specifically bind to two different antigens or two distinct epitopes of the same antigen either simultaneously or sequentially, introducing a new era of tumor immunotherapy (1, 2). Cadonilimab is the first dual immune checkpoint inhibitor (ICI) for tumor treatment entirely developed and approved in China. This agent simultaneously targets two immune checkpoints, namely programmed cell death protein 1 (PD-1) and cytotoxic T lymphocyte antigen 4 (CTLA-4), enabling preferential binding to tumor-infiltrating lymphocytes (3). The results of clinical trials with this drug have demonstrated novel breakthroughs in the treatment of advanced cervical cancer (4), gastric cancer with low or negative programmed death ligand 1 (PD L1) expression (5), and lung cancer (AK104 IIT 018).

ICIs block T cell negative regulatory signals, relieve immunosuppression, and enhance T cell antitumor effects. However, they may also abnormally potentiate immune responses, leading to disruption of immune tolerance and immune-related adverse events (irAEs) that affect normal tissues. Among these, irAEs involving the skin, gastrointestinal tract, endocrine system, lungs, and musculoskeletal system are relatively common (6). Common adverse drug reactions (ADRs) of BsAbs in antitumor therapy include cytokine release syndrome, neurotoxicity, infection, and hematologic and lymphatic toxicities (7).

According to the literature, the combination of PD 1 and CTLA 4 antibodies is associated with a marked increase in grade 3–4 ADRs without necessarily improving clinical benefit (8). Although cadonilimab targets two immune checkpoints simultaneously, its safety profile in the process of enhancing immune killing effects warrants in depth investigation. Thus far, no case of severe multi organ injury induced by cadonilimab has been reported. In this article, we focus on the underlying mechanisms and strategies for the management of multi organ injury. The aim is to highlight that close monitoring of multi system organ function is essential when using BsAbs in clinical practice. Moreover, we propose that novel biomarkers should be utilized to improve early diagnosis and prognosis of multi organ injury, thereby enhancing the safety of this therapeutic approach.

## 2 Case presentation

In February 2022, a 53-year-old female patient presented with irregular vaginal bleeding without obvious cause. Human papillomavirus (HPV) testing was positive for HPV-16 and HPV-50. Cervical biopsy pathology showed non-keratinizing squamous cell carcinoma. On February 18, 2022, pelvic magnetic resonance imaging (MRI) revealed an irregular mass with isointense T1 and slightly hyperintense T2 signals in the cervix, measuring approximately 4.9 cm × 4.8 cm, consistent with cervical cancer. The patient was diagnosed with stage III C1r squamous cell carcinoma of the cervix and received two cycles of concurrent radiotherapy with nedaplatin and cisplatin, plus nimotuzumab targeted therapy. Subsequently, she received three cycles of albumin-bound paclitaxel plus cisplatin combined with nimotuzumab, with the last treatment completed in July 2022. The treatment response was evaluated as partial response.

On December 4, 2023, contrast-enhanced computed tomography of the neck, chest, and abdomen suggested possible enlarged lymph nodes. On December 18, 2023, lymph node biopsy showed metastatic squamous cell carcinoma, consistent with cervical origin based on the patient’s history. On December 30, 2023, brain MRI showed a left maxillary sinus cyst, with no abnormalities on cranial MRI; pathological analysis revealed a small amount of squamous cell carcinoma, also considered of cervical origin. Subsequently, the patient was treated with a TP regimen: albumin-bound paclitaxel 0.3 g (January 2, 2024) plus cisplatin 40 mg (January 2–4, 2024) plus cadonilimab 500 mg (January 2, 2024). On January 13, 2024, microsatellite instability testing of cervical tissue showed a microsatellite stable status. On January 16, 2024, PD L1 testing showed a Tumor cell Proportion Score of 45% and a Combined Positive Score of 46%. Following discharge, the patient reported intermittent wheezing and chest tightness worsening over 1 week, with occasional cough and sputum production. On January 31, 2024, she was admitted to our hospital primarily due to wheezing for more than half a month, worsening for 1 week, after radiotherapy and chemotherapy for cervical cancer with mediastinal lymph node metastasis.

### 2.1 Diagnosis and treatment

On January 31, 2024, laboratory examinations yielded critical results: cardiac troponin I (cTnI) 0.779 μg·L^−1^; C-reactive protein 11.80 mg·L^−1^; liver function, myocardial enzymes, and electrolytes: sodium 138.0 mmol·L^−1^, potassium 3.90 mmol·L^−1^, alanine aminotransferase (ALT) 427.0 U·L^−1^, aspartate aminotransferase (AST) 538.0 U·L^−1^, total bilirubin 9.1 μmol·L^−1^, direct bilirubin 3.8 μmol·L^−1^, glucose 6.82 mmol·L^−1^, creatine kinase (CK) 6,867.4 U·L^−1^, creatine kinase MB (CK MB) 195.55 U·L^−1^, lactate dehydrogenase 1,601 U·L^−1^, α hydroxybutyrate dehydrogenase 1,430.7 U·L^−1^; thyroid function tests: thyroid stimulating hormone 0.107 mIU·L^−1^, triiodothyronine 1.63 nmol·L^−1^, thyroxine 190.05 nmol·L^−1^, pituitary function was normal. The patient reported palpitations and shortness of breath with an oxygen saturation of 99%. Oxygen was administered via a nasal cannula, and palpitations improved. Electrocardiography (ECG) showed sinus tachycardia (121 bpm). Transthoracic echocardiography at rest revealed mild tricuspid regurgitation, minimal pericardial effusion, normal left ventricular systolic function, and reduced diastolic function. Initial diagnoses included grade 2 myocarditis, grade 3 liver injury, grade 1 pneumonitis, and grade 1 hyperthyroidism.

Treatment was initiated with methylprednisolone sodium succinate 120 mg intravenously once daily (ivgtt qd) from January 31 to February 7, 2024, as pulse therapy; the treatment also included magnesium isoglycyrrhizinate 200 mg ivgtt qd from January 31 to February 8, plus polyene phosphatidylcholine injection 465 mg ivgtt qd from February 1 to February 8 for liver protection; and ceftriaxone sodium 2 g ivgtt qd from January 31 to February 8 for anti infection. On February 1, 2024, the patient reported relief of chest tightness and wheezing. On February 2 and February 7, the patient underwent further examination of liver function, renal function, myocardial enzymes, N terminal pro B type natriuretic peptide, and cTnI levels (**Table 1**). Methylprednisolone was tapered to 80 mg ivgtt qd from February 7 to February 10. The symptoms improved markedly, and the levels of liver enzymes and myocardial enzymes decreased, indicating effective treatment. She was discharged on February 11, 2024, receiving oral methylprednisolone 64 mg once daily.

**Table 1:** Changes in laboratory indicators.

Laboratory indicator	31-01-2024	01-02-2024	02-02-2024	04-02-2024	07-02-2024	23-04-2024
Cardiac Troponin I(μg·L^-1^)	0.779	0.459	0.469	0.454	0.259	0.012
C-reactive protein(g·L^-1^)	11.8	/	/	1. 19	/	/
Alanine Aminotransferase(U·L^-1^)	427	348	276	222.2	126	13.9
Aspartate Aminotransferase(L^-1^)	538	282	135.3	87.3	49.6	17.2
Total Bilirubin(μmol·L^-1^)	9.1	/	/	6.6	7.2	/
Direct Bilirubin(mol·L^-1^)	3.8	/	/	3.6	3.3	/
Creatine Kinase(U·L^-1^)	6 867.4	3 418	1 676.5	1 165.8	617.4	/
Creatine kinase-MB(U·L^-1^)	195.55	83.13	45.36	45.4	49.91	/
Lactate dehydrogenase(U·L^-1^)	1 601	1 309	1 110	1 037	779	/
Hydroxybutyrate-dehydrogenase(U·L^-1^)	1 430.7	1 235.1	1 094.1	1 011.3	744.5	/
Thyroid Stimulating Hormone(mIU·L^-1^)	0.107	/	/	0.059	/	1.342
Triiodothyronine(nmol·L_-1_)	1.63	/	/	1.02	/	2.25
Thyroxine(mo1·L^-1^)	1 90.05	/	/	168.64	/	116.35
N-terminal pro-brain natriuretic peptide(ng·L^-1^)	/	159	245	/	/	26.9

”/”indicates no data.

### 2.2 Post-discharge follow-up

On February 8, 2024, chest computed tomography showed enlarged mediastinal lymph nodes, with no significant change observed compared with February 1, 2024. The patient was discharged on February 11 receiving oral methylprednisolone 64 mg once daily. After discharge, the dose was reduced by 8 mg every 5 days (February 15, February 20, February 25, March 1, March 6, March 11, and March 16). It reached 8 mg/day on March 16, was further reduced to 4 mg/day on March 22, and was discontinued on March 27. Myocardial enzymes, liver enzymes, thyroid function, and blood glucose were monitored throughout the taper. Cadonilimab was permanently discontinued. On April 23, 2024, anticancer therapy was changed to albumin-bound paclitaxel 0.3 g plus bevacizumab 400 mg. Brain and abdominal MRI on April 26 showed multiple small hepatic cysts and left maxillary sinus inflammation, with no intracranial abnormality; the tumor response remained a partial response. At the 6-month follow-up after discharge, the cardiac injury, liver injury, and endocrine toxicity had resolved, the relevant laboratory indices had returned to their reference ranges (**Table 1**), and no residual symptoms, complications, or need for additional immunosuppressive treatment were reported. The patient remained under oncology follow-up while receiving the revised anticancer regimen. The chronological relationship among drug exposure, symptoms, investigations, treatment, and outcomes is summarized in **Table 2** and **Figure 1**.

**Table 2:** Integrated clinical timeline of treatment exposure, manifestations, investigations, management, and outcomes.

Date	Anticancer/immune therapy	Clinical signs and symptoms	Relevant tests/laboratory findings	Management and outcome
18 Feb 2022	Diagnosis; no immunotherapy	Irregular vaginal bleeding	Pelvic MRI: 4.9 × 4.8 cm cervical mass; biopsy: non-keratinizing squamous cell carcinoma; stage IIIC1r	Cervical cancer diagnosed
Feb–Jul 2022	Concurrent radiotherapy + nedaplatin/cisplatin + nimotuzumab; then nab-paclitaxel/cisplatin + nimotuzumab	—	Imaging response assessment	Partial response
4–30 Dec 2023	—	Recurrent disease suspected	Enlarged lymph nodes; biopsy confirmed metastatic squamous cell carcinoma of cervical origin; brain MRI without intracranial abnormality	Systemic treatment planned
2–4 Jan 2024	Nab-paclitaxel 0.3 g (2 Jan) + cisplatin 40 mg/day (2–4 Jan) + cadonilimab 500 mg (2 Jan)	Initially no acute symptoms	MSS; PD-L1 TPS 45%, CPS 46%	Single documented cadonilimab administration
Mid–31 Jan 2024	Cadonilimab not re-administered	Intermittent dyspnea and chest tightness for >2 weeks, worsening for 1 week; palpitations; occasional cough/sputum	ECG: sinus tachycardia 121 bpm; cTnI 0.779 μg/L; CK 6,867.4 U/L; CK-MB 195.55 U/L; ALT 427 U/L; AST 538 U/L; TSH 0.107 mIU/L; echocardiography: minimal pericardial effusion with preserved LV systolic function	G2 myocarditis, G3 liver injury, G1 pneumonitis, and G1 hyperthyroidism diagnosed
31 Jan–11 Feb 2024	Cadonilimab withheld	Chest tightness and dyspnea improved	Progressive decline in cardiac and hepatic injury markers (**Table 1**)	IV methylprednisolone 120 mg/day (31 Jan–7 Feb), then 80 mg/day (7–10 Feb); liver-supportive therapy and ceftriaxone; discharged 11 Feb on oral methylprednisolone 64 mg/day
15 Feb–27 Mar 2024	No further cadonilimab	No recurrent reported cardiopulmonary symptoms	Serial cardiac enzymes, liver enzymes, thyroid function, and glucose monitored	Oral methylprednisolone reduced by 8 mg every 5 days; 8 mg on 16 Mar, 4 mg on 22 Mar, discontinued 27 Mar
23 Apr–Aug 2024	Nab-paclitaxel + bevacizumab; cadonilimab permanently discontinued	No residual symptoms or complications reported at 6 months	Cardiac, hepatic, and endocrine laboratory indices returned to normal ranges; imaging showed maintained partial response	Toxicities resolved without additional immunosuppression; ongoing oncology follow-up

**Figure 1:**
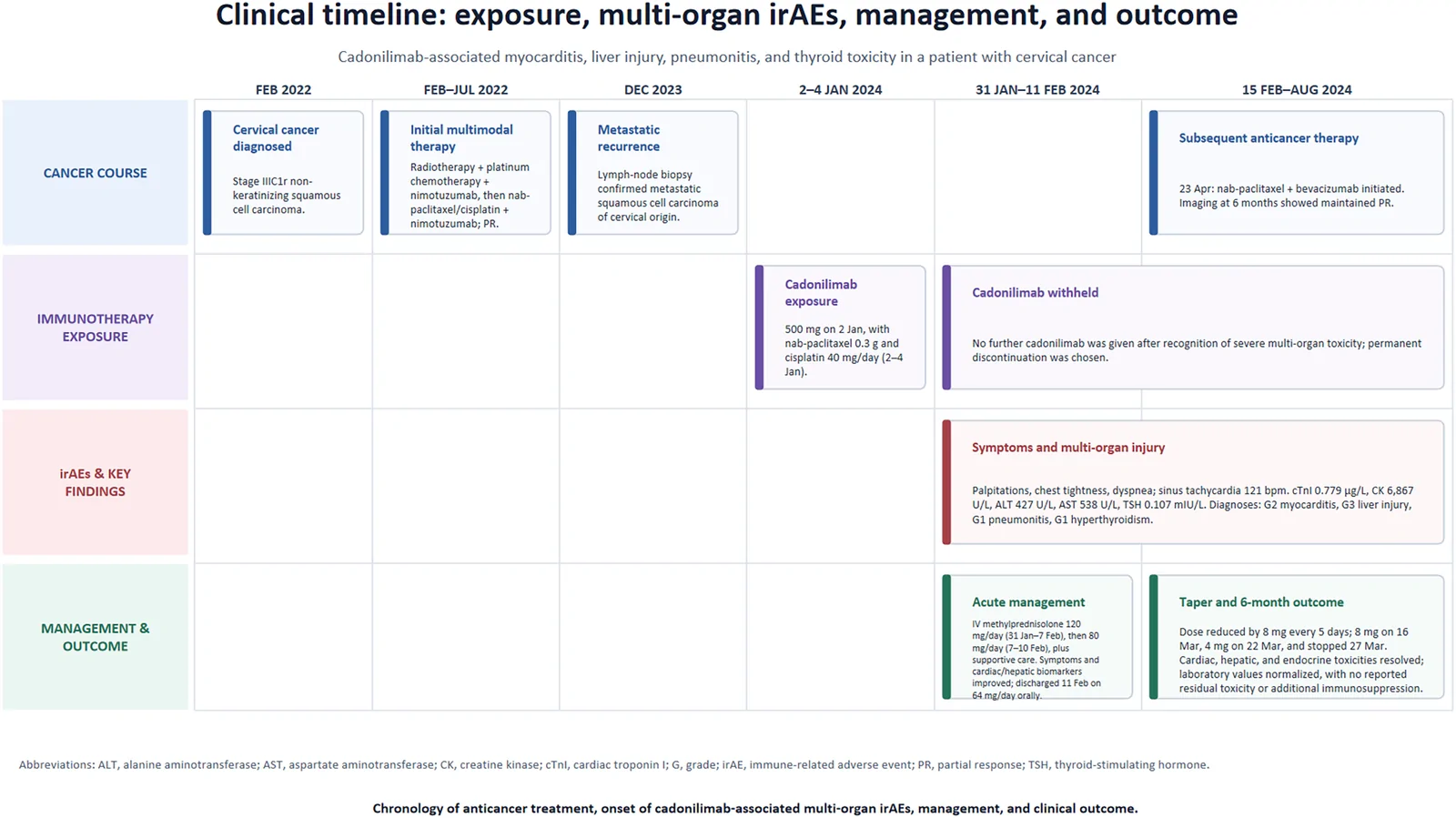
Chronology of anticancer treatment, onset of cadonilimab-associated multi-organ immune-related adverse events, management, and clinical outcome. ALT, alanine aminotransferase; AST, aspartate aminotransferase; CK, creatine kinase; cTnI, cardiac troponin I; G, grade; irAE, immune-related adverse event; PR, partial response; TSH, thyroid-stimulating hormone.

### 2.3 Ethics approval and consent to participate

The study was approved by the Clinical Research Ethics Committee of the Fourth Hospital of Hebei Medical University (approval number: 2023-126-K92). The patient was informed regarding the study protocol and provided written informed consent to participate in the study.

## 3 Discussion

### 3.1 Characteristics of ICI-induced multi-organ injury

ICI-related cardiotoxicity manifests variably as myocarditis, arrhythmias, conduction disorders, and myocardial infarction. ICI-associated myocarditis is a rare (incidence ≤1%) but severe ADR with a median onset of 17–65 days post-treatment (9–12) and a high mortality rate of 38% (12). The patient had no prior cardiovascular disease, while baseline cardiac markers and ECG were normal. She developed intermittent wheezing and chest tightness 14 days after receiving TP plus cadonilimab, with cTnI, CK, and CK-MB levels markedly exceeding the upper limit of normal (ULN); the onset of symptoms was earlier than that previously reported in the literature. ECG showed no features of acute coronary syndrome; together with other cardiac markers, the findings met the diagnostic criteria for “suspected myocarditis”, graded as G2 myocardial injury (9–11, 13–15).

Immunotherapy-mediated hepatotoxicity (IMH) typically occurs within 4–12 weeks after the first treatment and is the third most common irAE. With monotherapy, elevation of ALT/AST levels occurs in 3–9% of patients receiving CTLA-4 inhibitors, 1.5–5% receiving PD-1 inhibitors, and 3% receiving PD-L1 inhibitors, with grade 3 or 4 severe hepatotoxicity accounting for approximately 1% of cases (16). The onset in this case was earlier than that reported in the literature. According to the Common Terminology Criteria for Adverse Events (CTCAE) version 6 (17), the ALT/AST levels of this patient were 5–10-fold higher than the ULN after treatment with cadonilimab, and this condition was classified as grade 3 liver injury (ALT or AST > 5 × ULN to < 20 × ULN). Based on the Chinese Guidelines for the Diagnosis and Management of Drug-Induced Liver Injury (2023 Edition) (18), the Roussel Uclaf Causality Assessment Method scale score was 7, indicating that the liver injury was likely induced by the drug. The R value was 3.8 (2 < R < 5), consistent with a mixed clinical phenotype. Next, the patient received albumin-bound paclitaxel without liver injury, temporarily excluding paclitaxel as the cause.

ICI-related hyperthyroidism typically occurs within 2–6 weeks after treatment (13, 19). The patient had normal baseline thyroid function and exhibited free thyroxine levels markedly exceeding the ULN after receiving TP plus cadonilimab; the onset was consistent with that previously reported in the literature. According to CTCAE version 6 (17), the thyroid toxicity was grade 2 (symptomatic; thyroid hormone replacement therapy indicated).

Comparisons of ADR incidence among patients receiving PD-1/PD-L1 inhibitors, CTLA-4 inhibitors, and PD-1/CTLA-4 BsAb combination therapy are summarized in **Table 3** (12, 13, 20–22). A meta-analysis showed that dual ICIs (PD-1 plus CTLA-4 inhibitors) did not increase overall cardiovascular adverse events compared with monotherapy; nonetheless, the incidence of myocarditis was significantly higher in the combination group (12). According to these studies, CTLA-4 inhibitors generally increase the incidence of IMH more than PD-1/PD-L1 inhibitors, and ICI combination therapy further increases the risk of IMH. Additionally, elevated baseline AST levels, pre-existing autoimmune disease, and history of liver disease may also increase the risk of IMH (16, 23, 24). Cadonilimab has been on the market for a short time and was not included in these studies; however, based on the above evidence, the incidence rates of cadonilimab-related myocarditis, immune-mediated hepatitis, and immune-related hyperthyroidism are expected to be higher than those associated with monospecific antibody immunotherapy.

**Table 3:** Summary of the incidence rates of immune-related adverse reactions of different ICIs (12, 13, 20–22)(%).

Drug classification	ICIs	Myocarditis incidence (%)	Grade 3–4 myocarditis incidence (%)	Hepatotoxicity incidence (%)	Grade 3–4 hepatotoxicity incidence (%)	Hyperthyroidism incidence (%)
PD-L1	Atezolizumab	0.13	0.05	9	2.9	/
	Durvalumab	0.16	0.06	12	4.8	/
	Avelumab	0.00	0.00	0.9	0.6	2.3
PD-1	Pembrolizumab	0.22	0.13	0.7	0.14	3.7
	Nivolumab	0.15	0.04	1.8	/	2.8
	Cemiplimab	0.00	0.12	2.1	1.9	/
CTLA-4	Ipilimumab	0.04	0.04	4.5	2	1.4
PD-1 + CTLA-4	Nivolumab + Ipilimumab	0.34	0.16	13	/	9.4
	Pembrolizumab + Ipilimumab	/	/	/	/	10.4

”/”indicates no data.

According to five major guidelines on the management of ICI toxicity issued by the Chinese Society of Clinical Oncology, European Society for Medical Oncology, Society for Immunotherapy of Cancer, National Comprehensive Cancer Network, and American Society of Clinical Oncology (25–29), the occurrence of CTCAE grade 3–4 ADRs generally necessitates discontinuation of ICIs. For grade 3 ADRs, after symptoms and/or laboratory parameters improve to grade 1 or lower, administration of ICIs may be cautiously resumed. The management of grade 4 ADRs requires permanent discontinuation of therapy with ICIs. Management options include ICI withdrawal, symptom treatment, corticosteroids, immunosuppressants, and hemodialysis. Moreover, the use of immunosuppressants for irAEs does not appear to compromise the antitumor activity of ICIs (30). The present patient developed concurrent grade 2 myocarditis, grade 3 liver injury, and grade 2 thyroid toxicity. The clinical pharmacist recommended permanent discontinuation of cadonilimab and cautious use of other ICIs, taking into account future oncologic needs. Following ICI withdrawal, the patient did not experience tumor progression. For steroid-refractory or rapidly progressive toxicity, escalation should be individualized by the organ involved and guided by a multidisciplinary team. Potential options include mycophenolate mofetil or tacrolimus for immune-mediated hepatitis and mycophenolate mofetil, intravenous immunoglobulin, anti-thymocyte globulin, abatacept, or other targeted immunomodulation for myocarditis; infliximab should be avoided when significant hepatic injury is present. None of these second-line agents was required in this patient because symptoms and laboratory abnormalities improved promptly with corticosteroids and supportive therapy.

### 3.2 Literature review

#### 3.2.1 Cases and characteristics of multi-organ injury induced by cadonilimab

Cadonilimab is a PD-1/CTLA-4 BsAb with a unique “four-leaf clover” structure independently developed and currently only approved in China. A previous study (31) included 19 reports involving 464 patients with 661 ADRs, mainly affecting the blood/lymphatic system (31.62%), gastrointestinal system (16.49%), hepatobiliary system (13.92%), general disorders and administration site conditions (11.04%), endocrine system (8.17%), and skin/appendages (6.05%). Among these, there were 86 cases of IMH (13%) and five cases of immune-related myocarditis (0.76%); no hyperthyroidism cases were reported. Treatment for cadonilimab-related liver injury mainly includes corticosteroids, mycophenolate mofetil, and tacrolimus. In case corticosteroids are ineffective, anti-thymocyte globulin may be used. Monoclonal antibodies against interleukin-6 (IL-6), CD20, and tumor necrosis factor-alpha (TNF-α) have also been successfully used (32). Due to the risk of hepatotoxicity, infliximab should not be used in patients with liver injury (33). Although immune-related myocarditis induced by cadonilimab is rare (incidence <1%), it is a severe irAE with a mortality rate as high as 36.7% (34). Approximately 94% of patients with ICI-related myocarditis have elevated troponin levels. When only cardiac biomarkers are elevated, patients may be asymptomatic; nevertheless, myocarditis can suddenly become fulminant, leading to cardiogenic shock or death (35). A previous study (36) reported a case of sudden cardiac death in a patient receiving cadonilimab plus anlotinib (a small-molecule multi-target tyrosine kinase inhibitor with known cardiovascular toxicity). The combination may enhance cardiotoxicity, especially when the cadonilimab dose (15 mg/kg every 3 weeks) exceeds the standard dose (10 mg/kg every 3 weeks). This suggests that when cadonilimab is combined with anti-angiogenic agents, strict adherence to the standard dose and cardiac monitoring are essential.

In four clinical studies of monotherapy with cadonilimab (COMPASSION-01; COMPASSION-03; AK104-202; COMPASSION-06; total: 461 patients) (37–40), 340 patients received 6 mg/kg every 2 weeks, while 121 patients received other doses. Immune-related myocarditis occurred in three patients (0.7%)(grade≥3: 2 [0.4%]). Immune-related hepatitis occurred in 3 patients (0.7%) (all cases were grade 3). Immune-related hyperthyroidism occurred in 23 patients (5.0%)(grade≥3: 3 [0.7%]). Clinical trial data are summarized in **Table 4**.

**Table 4:** Clinical trials related to cadonilimab.

Identifier	Tumor	Line	Drug	Phase	Number	ORR(%)	DCR(%)	PFS (m)	OS (m)	Number of Immune related myocarditis	Number of Immune related hepatitis	Number of Immune-related hyperthyroidism
COMPASSION-01 (37)	Advanced or metastatic solid tumor	≥2	Cadonilimab 0.2–2;4;6;10 mg/kg q2w or 450 mg q2w or 15/25 mg/kg q3w	phase 1a/1b	119(13;3;37;18;18;12;18)	13.4	NA	5.5	15.6	0	2 vs 2^a^	12 vs 1^a^
COMPASSION-03 (38)	Unresectable advanced solid tumors	≥2	Cadonilimab 6/10 mg/kg or 450 mg q2w	phase 1b/2	240(197;43)	13.30%	NA	NA	NA	3 vs 2^a^	1 vs 1^a^	5 vs 2^a^
AK104–202 (39)	Advanced NSCLC	≥ 2	Cadonilimab 6 mg/kg q2w	phase 1b/2	53	5.70%	35.80%	1.9	13.3	0	0	6
COMPASSION-06 (40)	Recurrent or metastasis nasopharyngeal carcinoma	3	Cadonilimab 6 mg/kg q2w	phase 1b/2	23	26.10%	56.50%	3.71	NR	0	0	0

^a^Grade 3 or above adverse reactions; **NA**, not available; NR, not reached.

#### 3.2.2 Mechanisms of ICI-induced irAEs

A meta-analysis of 44 ICI-related randomized controlled trials (n=23,759) showed substantial heterogeneity in irAE incidence across trials. Specifically, the rates of any-grade irAEs ranged from 16.9% to 96% (median: 28.5%), while those of grade 3–4 irAEs ranged from 2% to 23% (median: 7.5%). PD-L1 and CTLA-4 inhibitors have distinct irAE profiles. PD-L1 inhibitors commonly cause hypothyroidism, pneumonitis, myocarditis, arthralgia, and autoimmune diabetes; CTLA-4 inhibitors commonly cause hypophysitis, dermatitis, and enteritis/colitis (41). Studies suggest that CTLA-4 primarily acts at the early stage of immune responses (42), whereas PD-1 mainly acts at the later stage (43). The authors hypothesized that this difference underlies the distinct irAE profiles of these two classes. Consequently, BsAbs, which block both pathways, may theoretically induce irAEs in a broader range of organs compared with monospecific antibodies.

Based on their mechanism of action, ICI toxicities can affect any organ exposed to environmental antigens or mutagens (e.g., thyroid, heart, and gastrointestinal tract), often presenting as inflammation resembling autoimmune diseases. The mechanisms underlying ICI-induced irAEs are discussed below (44, 45) (**Figure 2**):

**Figure 2:**
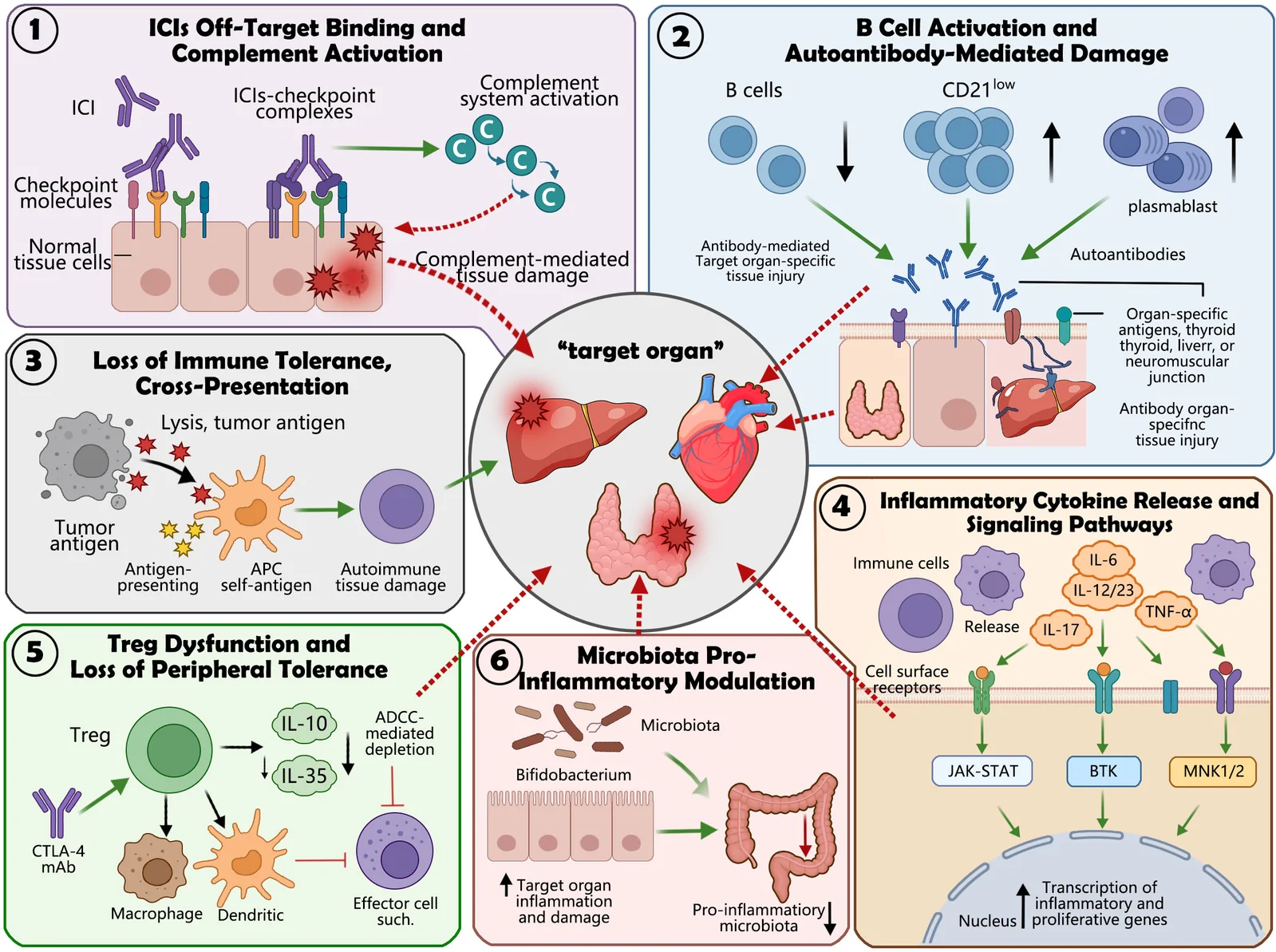
The mechanisms underlying ICI induced irAEs.

Firstly, off-target effects. Immune checkpoints are expressed on normal human tissues. ICIs directly bind to them, inducing complement activation and causing damage to the bound normal tissues via the complement system.

Secondly, disruption of immune tolerance. ICIs break the original immune tolerance balance, enhancing autoimmunity and triggering irAEs. Shared antigens exist between self-antigens and tumor antigens. Tumor cells release host antigens during treatment, leading to antigen cross-presentation and subsequent autoimmune responses.

Thirdly, B-cell activation and antibody-mediated damage. ICI combination therapy induces early B-cell changes, including decreased circulating B cells, increased CD21+ subsets, and increased plasmablasts. Autoantibodies have been detected in numerous patients with irAEs, such as in those with immune-related thyroiditis, myasthenia gravis, and type 1 diabetes.

Fourthly, inflammatory mediators released by immune cells. During the occurrence of irAEs, the levels of various inflammatory cytokines (e.g., IL-1, IL-6, IL-12/23, IL-17, TNF-α) are elevated. These cytokines mediate tissue damage and bind to receptors on lymphocytes or innate immune cells, thereby activating downstream signaling pathways (e.g., Janus kinase-signal transducer and activator of transcription [JAK-STAT], Bruton tyrosine kinase [BTK], MAP kinase interacting serine/threonine kinase 1/2 [MNK1/2]) and promoting the transcription of genes encoding proliferative and inflammatory proteins.

Fifthly, pro-inflammatory effects of the microbiota. The gut microbiota can exacerbate pro-inflammatory responses during ICI therapy, thus triggering irAEs. Studies involving mouse models showed that administration of Bifidobacterium can reduce CTLA-4-induced colitis weight loss without affecting the efficacy of ICIs.

Finally, loss of peripheral tolerance due to dysfunction of immune regulatory cells. Regulatory T cells (Tregs) are crucial for maintaining immune homeostasis. They modulate immune responses by secreting anti-inflammatory cytokines (e.g., IL-10, IL-35) and influencing macrophages and dendritic cells. CTLA-4 antibodies can induce Treg depletion via antibody-dependent cell-mediated cytotoxicity, whereas the effect of PD-1 antibodies on Tregs remains controversial. Based on the above, BsAb-induced irAEs likely involve multiple mechanisms.

Deep sequencing of the T cell receptor β chain complementarity-determining region 3 in patients with fatal myocarditis revealed high overlap between T-cell clones in the heart and tumor-infiltrating lymphocytes, indicating that T-cell-mediated cross-antigen reaction plays a critical role in myocarditis. The histopathology of IMH can be classified into hepatocyte-dominant or bile duct-dominant injury. Hepatocyte-dominant injury shows mixed panlobular immune cell infiltration, predominantly lymphocytes, with focal necrosis. Bile duct injury mainly presents as bile duct proliferation and mixed portal inflammation, with rare lobular necroinflammation (45). The mechanism of ICI-induced thyroiditis is typically a destructive course; it involves an initial release of preformed thyroid hormones (thyrotoxic phase), followed by thyroid destruction and insufficient hormone production leading to hypothyroidism, which is often permanent. The transition from thyrotoxicosis to hypothyroidism may occur within days to weeks, whereas in classic thyroiditis this process lasts for weeks to months. The proposed pathophysiology involves lymphocytic and histiocytic infiltration of the thyroid (45, 46). Studies showed that immune-related thyroid dysfunction is closely associated with autoimmune antibody-mediated damage; elevated levels of thyroid peroxidase and thyroglobulin antibodies were detected in numerous patients with ICI-related hypothyroidism (45). Unfortunately, sequencing and thyroid peroxidase/thyroglobulin antibody testing were not performed in this case. With the application of new technologies (e.g., proteomics, genomics, and toxicometabolomics), novel biomarkers (e.g., IL-1, IL-6, IL-12/23, IL-17, TNF-α) continue to emerge, playing important roles in early diagnosis and the prediction of irAEs. In this patient, no cytokine panel, immune-cell profiling, T-cell receptor clonality analysis, single-cell RNA sequencing, or thyroid peroxidase/thyroglobulin antibody testing was performed. Accordingly, the pathways discussed above represent biologically plausible mechanisms extrapolated from the broader ICI literature rather than mechanisms demonstrated in this individual patient. Prospective collection of paired blood and, when clinically justified, tissue samples will be required to test these hypotheses and identify predictive or pharmacodynamic biomarkers of cadonilimab-related irAEs.

Retrospective observational studies have identified risk factors for higher irAE incidence, including clinical characteristics [e.g., female sex (47)], germline and somatic genetic features (48), specific microbiota composition [e.g., Gram-negative bacteria associated with inflammatory host gut gene signatures and poor prognosis (49)], and circulating biomarkers (50). Additionally, ICIs may be combined or sequenced with other systemic therapies (e.g., chemotherapy, targeted therapy) or other modalities (e.g., radiotherapy, allogeneic stem cell transplantation). Such combinations have distinct toxicity profiles (51). Hence, physicians should evaluate the toxicities associated with each agent and remain vigilant for the occurrence of novel toxicities.

Studies have shown that patients who develop irAEs often have better treatment outcomes [e.g., improved response rates and prolonged survival (52)] or similar outcomes (53) compared with those without irAEs. Most clinicians recognize that although irAEs are associated with treatment toxicity, their occurrence also indicates sufficient immune system activation, which may enhance tumor targeting. Therefore, irAE management aims to provide the minimum immunosuppression needed to control symptoms (54). For certain patients, such as those with immune-mediated endocrinopathies, asymptomatic hepatitis, or pancreatitis, treatment interruption without immunosuppression may be appropriate. Managing irAEs often requires the use of immunosuppressive drugs (e.g., corticosteroids), which may attenuate ICI efficacy. Given this trade-off, understanding the cellular and molecular targets involved in irAEs could enable targeted management of specific immune cells and molecular pathways, potentially treating irAEs while preserving or even enhancing ICI efficacy. Studies have identified potential cellular and molecular targets during ICI therapy, including T-cell subsets, myeloid subsets, B cells, and integrins. Intervening in these targets may help alleviate irAEs without compromising the antitumor activity of ICIs. The current research further proposes collecting blood and tissue samples at irAE onset for multi-omics analysis to identify predominant inflammatory patterns, selecting targeted therapy based on individual inflammatory signatures, and monitoring clinical responses to such therapy. This evidence highlights the potential of precision medicine in managing irAEs (55).

This report has several limitations. First, it describes a single patient and therefore cannot establish incidence, comparative risk, causality beyond a case-level assessment, or generalizability to other populations. Second, the retrospective literature synthesis is vulnerable to publication bias, selective outcome reporting, heterogeneous adverse-event definitions, and differences in follow-up and treatment exposure; therefore, cross-study incidence comparisons should be interpreted as descriptive rather than definitive. Third, case-specific molecular and immunological data were unavailable, and follow-up was limited to 6 months after discharge. Larger prospective multicenter cohorts are needed to define the incidence and risk factors of severe cadonilimab-related irAEs. Future studies should incorporate standardized longitudinal monitoring, harmonized toxicity definitions, immune profiling and multi-omics biomarker analyses, and prospective evaluation of organ-directed immunosuppressive strategies that control toxicity while preserving antitumor activity.

## 4 Conclusion

Severe multi-organ irAEs related to treatment with cadonilimab are rarely reported. This case demonstrates the need for early recognition, coordinated organ-specific assessment, prompt corticosteroid treatment, and a documented taper with longitudinal monitoring. Our descriptive literature review highlights potential differences in myocardial, hepatic, and thyroid toxicity profiles across ICI strategies, but it does not permit definitive comparative incidence estimates. Baseline assessment and active monitoring should accompany cadonilimab treatment. Larger multicenter studies with standardized follow-up are needed to determine incidence and risk factors, while prospective immune profiling and biomarker studies should clarify mechanisms and support early prediction. Evaluation of targeted immunosuppressive approaches may help control irAEs while preserving antitumor efficacy.

## Data Availability

The original contributions presented in the study are included in the article/supplementary material. Further inquiries can be directed to the corresponding author.
